# Associations between corneal biomechanical parameters and retinal superficial vessel density in young adults with varying myopia severity: a cross-sectional study

**DOI:** 10.3389/fmed.2026.1951319

**Published:** 2026-10-13

**Authors:** Yanzhi Guo, Xiaohong Liu, Yiwei Wang, Yang Zhang, Siqi He, Yumeng Zhang, Junjue Chen, Jili Chen, Yisheng Zhong, Huaping Liao

**Affiliations:** 1 Department of Ophthalmology, Ruijin Hospital Affiliated Medical School, Shanghai Jiaotong University, Shanghai, China; 2 Department of Ophthalmology, Shibei Hospital, Shanghai, China

**Keywords:** association, corneal biomechanical properties, Corvis ST, myopia, OCTA

## Abstract

**Aim:**

To evaluate associations between corneal biomechanical parameters and superficial retinal vessel density (SVD) in young adults with varying myopia severity and determine whether these associations differ myopia severity groups.

**Methods:**

This cross-sectional study included 167 participants contributing 320 eyes (93 low-, 125 moderate-, and 102 high-myopia eyes). Corneal biomechanical parameters were assessed using Corneal Visualization Scheimpflug Technology (Corvis ST), and macular and peripapillary SVD using optical coherence tomography angiography (OCTA). Eye-level analyses used linear mixed-effects models (LMMs) with participant identity as a random intercept to account for inter-eye correlation. Association models were adjusted for axial length, age, sex, and biomechanically corrected intraocular pressure (bIOP). Biomechanical parameter × myopia severity interactions were tested to assess between-group differences in associations.

**Results:**

Peak distance (PD), stress-strain index (SSI), stiffness parameter at first applanation (SP-A1), and Ambrosio relational thickness-horizontal (ARTh) differed significantly among myopia groups (all *P* < 0.05). Macular SVD differed in the inner nasal (IN) and outer nasal (ON) regions, while peripapillary SVD differed in the IN, inner temporal (IT), outer superior (OS), outer inferior (OI), ON, and outer temporal (OT) regions (all *P* < 0.05). After covariate adjustment, several corneal biomechanical parameters remained associated with regional SVD. IRA × myopia severity interactions were significant for central, II, IN, IT, and OT macular SVD (all *P* for interaction < 0.05), while PD also showed a significant interaction with myopia severity for central SVD (*P* for interaction = 0.023). For peripapillary SVD, a significant IRA × myopia severity interaction was observed in the OS region (*P* for interaction = 0.015).

**Conclusion:**

Corneal biomechanical parameters and retinal SVD showed regional differences across myopia severity groups. After accounting for inter-eye correlation and relevant covariates, selected biomechanical parameters were associated with regional SVD, and some associations differed by myopia severity. These findings suggest a potential relationship between anterior segment biomechanical characteristics and retinal microvascular alterations; however, the cross-sectional design precludes causal or longitudinal interpretation.

## 1 Introduction

The global prevalence of myopia has been rising steadily, with projections indicating that approximately 740 million children and adolescents will be affected by 2050 (1–4). High myopia, in particular, substantially elevates the risk of vision-threatening complications—including retinal detachment, retinoschisis, macular holes, and glaucoma—which may ultimately lead to irreversible visual impairment (5–7). Given that myopia is associated with structural differences in the anterior and posterior segments of the eye, simultaneously evaluating corneal biomechanical characteristics and retinal microvascular measurements may provide a more integrated understanding of myopia-related ocular alterations (8, 9). Consequently, investigating the relationship between anterior ocular biomechanical characteristics and posterior retinal microvascular measurements may improve our understanding of structural and microvascular alterations associated with myopia.

The pathogenesis of myopia remains incompletely understood, with current evidence supporting a multifactorial interplay between genetic predisposition and environmental influences (such as indoor spatial frequency characteristics and increased screen use) (10–13). Previous studies have reported differences in Corvis ST-derived dynamic corneal response parameters in myopic eyes, suggesting that ocular biomechanical responses may vary with refractive status and ocular geometry (14–16). Concurrently, optical coherence tomography angiography (OCTA) based investigations have consistently demonstrated that macular and peripapillary superficial vessel density progressively decreases with axial elongation and higher myopia severity (17–21). However, these two lines of evidence have largely evolved in parallel (22, 23). Direct investigations linking corneal biomechanical parameters to retinal microvascular density within the same myopic cohort remain scarce, and it remains unknown whether such associations—if present—are consistent across different myopia severities or exhibit stage-dependent patterns. This knowledge gap limits our understanding of the potential relationship between anterior ocular biomechanical characteristics and posterior retinal microvascular alterations in myopic eyes.

This study aims to investigate the associations between Corvis ST-derived corneal biomechanical parameters and retinal SVD in young adults with varying degrees of myopia and to determine whether these associations differed according to myopia severity. We hypothesize that selected corneal biomechanical parameters would be associated with regional retinal SVD and that these associations might differ across myopia severity groups. These findings may provide further insight into the relationship between anterior ocular biomechanical characteristics and retinal microvascular alterations in myopic eyes.

## 2 Materials and methods

### 2.1 Ethical approval

This study adhered to the tenets of the Declaration of Helsinki. The research protocol was reviewed and approved by the Institutional Review Board of Ruijin Hospital Affiliated to Shanghai Jiao Tong University School of Medicine [approval No. (2024) 118]. All participants were fully informed of the study purpose, procedures, and potential risks and benefits prior to enrollment, and written informed consent was obtained from each participant before any study-related procedures were performed.

### 2.2 Study participants

This prospective study enrolled 167 young and middle-aged myopic patients from Ruijin Hospital Affiliated to Shanghai Jiao Tong University School of Medicine between May 2024 and December 2024. The inclusion criteria were: (1) age 18–35 years; (2) best-corrected visual acuity (BCVA) ≥ 1.0 with astigmatism ≤2.0 diopters (D); (3) intraocular pressure (IOP) ranged from 10 to 21 mmHg with an interocular difference of less than 5 mmHg; (4) appropriate contact lens discontinuation period (soft lenses ≥1week, rigid lenses ≥3 weeks, orthokeratology lenses ≥3 months); and (5) no active ocular pathology on slit-lamp examination. Key exclusion criteria comprised: (1) history of ocular surgery or trauma; (2) refractive media opacity or abnormal fixation; (3) other ocular diseases; (4) systemic conditions affecting ocular circulation; and (5) recent use (within 2 weeks) of medications affecting blood flow. The study protocol was approved by the Institutional Review Board of Ruijin Hospital and conducted in accordance with the tenets of the Declaration of Helsinki. Written informed consent was obtained from all participants prior to any study procedures.

### 2.3 Study design

Based on spherical equivalent (SE) measurements, the study cohort was stratified into three subgroups: low myopia group (−3.00 D < SE ≤ −0.50 D), moderate myopia group (−6.00 D < SE ≤ −3.00 D) and high myopia group (SE ≤ −6.00 D).

### 2.4 Ophthalmic examination

All enrolled participants underwent standardized eye examinations between 9:00 a.m. and 4:00 p.m., with specific examination items including: (1) Detailed anterior segment and fundus evaluation using slit-lamp microscopy; (2) Uncorrected visual acuity (UCVA) assessment with the Standard Logarithmic Visual Acuity Chart (GB11533-2011); (3) Refraction was performed using the KR-1 autorefractor (TOPCON, Tokyo, Japan) followed by subjective refinement to determine BCVA and SE; (4) IOP measurement via non-contact tonometry (TX-20, Canon, Japan); (5) Axial length (AL) biometry using optical coherence technology (IOL Master 900, Haag-Streit, Switzerland).

### 2.5 OCTA examination

This study measured superficial retinal vessel density (SVD) in all participants using the ZEISS CIRRUS HD-OCT 5000 system. To minimize motion artifacts caused by eye movements, the device was equipped with FastTrac™ proprietary eye-tracking technology, which significantly improved scanning speed and image acquisition stability, thereby ensuring high-quality fundus vascular imaging (24). All OCTA examinations were performed using a standardized acquisition protocol, and image quality was assessed immediately after acquisition. Scans with obvious motion artifacts or a signal strength score <8 were repeated. Participants whose OCTA images showed persistent obvious motion artifacts or whose signal strength remained <8 after repeated scanning were excluded from the final analysis. All images included in the analysis were further visually inspected for scan centration, motion artifacts, and segmentation accuracy before quantitative analysis. Automated segmentation boundaries were visually reviewed, and scans with evident segmentation errors affecting the analyzed retinal slab were corrected when possible or excluded if reliable correction could not be achieved. Thus, only OCTA images with a signal strength score ≥8 and without substantial motion or segmentation artifacts were included in the final quantitative analysis. For OCTA imaging, the Angiography 6  ×  6 mm scanning mode was selected to focus on the superficial retinal layer (from the inner limiting membrane to the inner plexiform layer) for analysis. Following ETDRS standards, the macular area was divided into 9 sectors: a central circle with diameter of 1 mm (C), inner ring (1–3 mm) subdivided into inner superior (IS), inner inferior (II), inner nasal (IN) and inner temporal (IT) quadrants, and outer ring (3–6 mm) divided into outer superior (OS), outer inferior (OI), outer nasal (ON) and outer temporal (OT) quadrants. The same partitioning scheme was applied to quantify peripapillary SVD across eight regions (IS, II, IN, IT, OS, OI, ON, and OT). The Early Treatment Diabetic Retinopathy Study (ETDRS) grid in both the macular and peripapillary regions are illustrated in Figure 1. All OCTA images were processed using FORUM software (ZEISS) for automated calculation of SVD.

**Figure 1:**
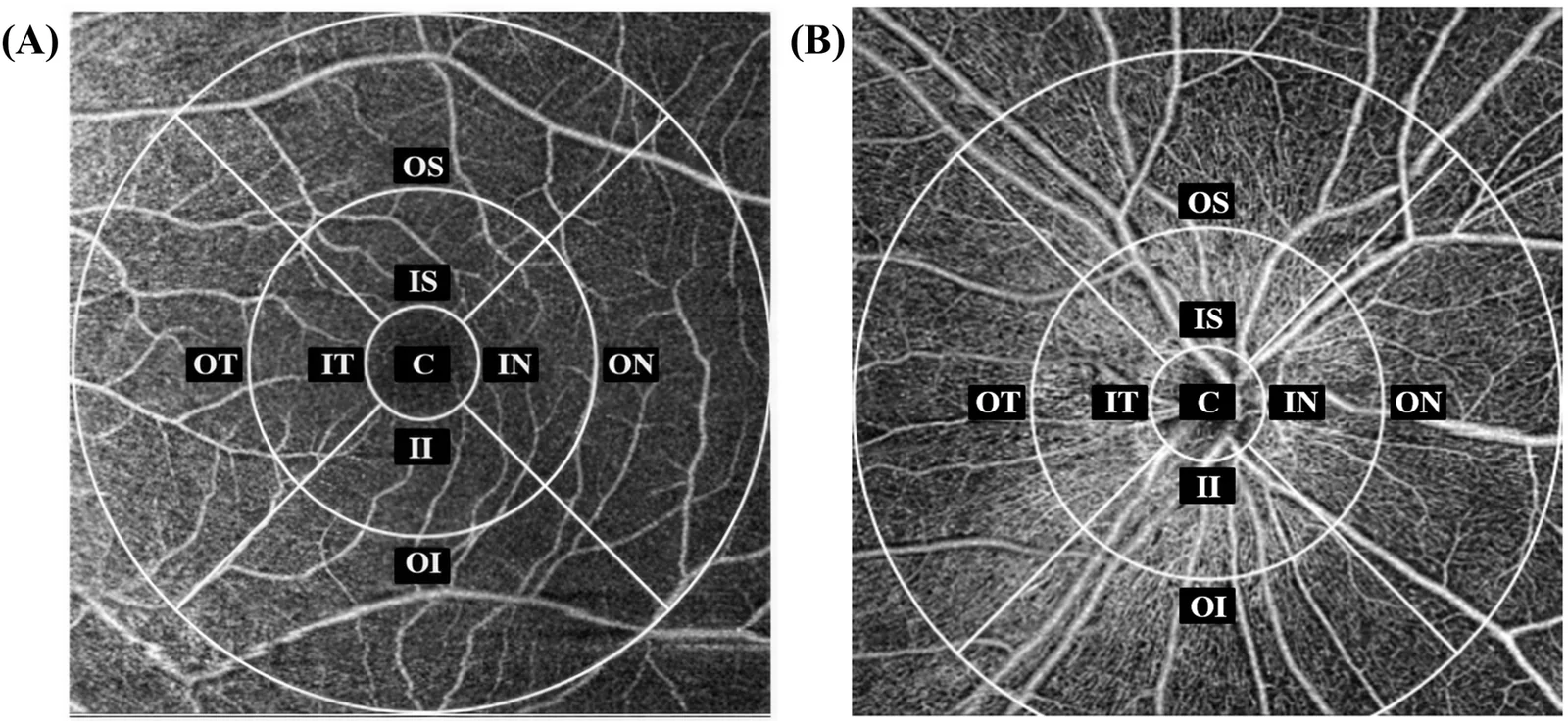
ETDRS grid partitioning schematic showing **(A)** macular area and **(B)** peripapillary region within 6 × 6 mm scan areas.

### 2.6 Corvis ST examination

This study assessed corneal biomechanical parameters in all participants using the Corvis ST tonometer (Oculus Optikgeräte GmbH, Wetzlar, Germany). The device employs a 455-nm wavelength LED light source and delivers an air pulse with a maximum pressure of 25 kPa to ensure measurement precision and reproducibility. Examinations were performed under natural pupil conditions without any mydriatic or miotic agents to maintain the eye's native physiological state. For each eye, three consecutive measurements were obtained, with the average value used for statistical analysis to ensure data reliability. Measurements failing quality control (indicated by yellow or red warnings) were repeated. Subjects with persistently unqualified measurements after three attempts were excluded from final data analysis.

Following each measurement, the Corvis ST automatically analyzes and generates a comprehensive set of corneal biomechanical parameters, including: (1) Time, length, and velocity from initiation to first applanation (First Applanation Time, AT1; First Applanation Length, AL1; First Applanation Velocity, AV1); (2) Time, length, and velocity from initiation to second applanation (Second Applanation Time, AT2; Second Applanation Length, AL2; Second Applanation Velocity, AV2); (3) Peak distance between maximum indentation points (Peak Distance, PD); (4) Maximum deformation amplitude (Deformation Amplitude, DA); (5) Deformation amplitude ratio (DA Ratio); (6) Integrated radius (IRA); (7) Stiffness Parameter at First Applanation (SP-A1), a dynamic response parameter derived from the relationship between the applied air-puff loading and corneal displacement at first applanation; (8) Ambrosio Relational Thickness-horizontal (ARTh), a pachymetric progression parameter; and (9) Stress-Strain Index (SSI), an algorithm-derived parameter developed to characterize the corneal material stress-strain behavior. A literature review revealed that 82.9% of corneal deformation parameters measured by Corvis ST demonstrated excellent measurement repeatability (25), supporting the reliability of this device.

### 2.7 Statistical analysis

All statistical analyses were performed using IBM SPSS Statistics version 27.0 (IBM Corp., Armonk, NY, USA). Continuous variables were summarized as mean ± standard deviation (SD) or median (interquartile range), as appropriate. Because both eyes from the same participant could be included, eye-level analyses were performed using linear mixed-effects models (LMMs), with participant identity included as a random intercept to account for inter-eye correlation. Models were estimated using restricted maximum likelihood (REML), and fixed effects were evaluated using Type III tests. For comparisons among the low-, moderate-, and high-myopia groups, myopia severity was included as a fixed effect, followed by Sidak-adjusted pairwise comparisons of estimated marginal means when appropriate. Associations between corneal biomechanical parameters and macular or peripapillary superficial vessel density (SVD) were assessed using multivariable LMMs adjusted for axial length (AL), age, sex, and biomechanically corrected intraocular pressure (bIOP). To assess whether these associations differed according to myopia severity, biomechanical parameter × myopia severity interaction terms were tested in the full-sample models, followed by stratified LMMs to characterize group-specific associations. Results are reported as unstandardized regression coefficients (*β*) with 95% confidence intervals (CIs) and exact *P* values. All tests were two-sided, with *P* < 0.05 considered statistically significant.

## 3 Results

### 3.1 Clinical characteristics of patients

A total of 167 participants contributing 320 eyes were included in the analysis (Figure 2). Among them, 153 participants contributed both eyes, whereas 14 participants contributed one eye only. The demographic and ocular characteristics of the low-, moderate-, and high-myopia groups are summarized in Table 1. After accounting for within-subject inter-eye correlation using LMMs with participant identity included as a random intercept, significant intergroup differences were observed in AL and IOP (both *P* < 0.05).

**Figure 2:**
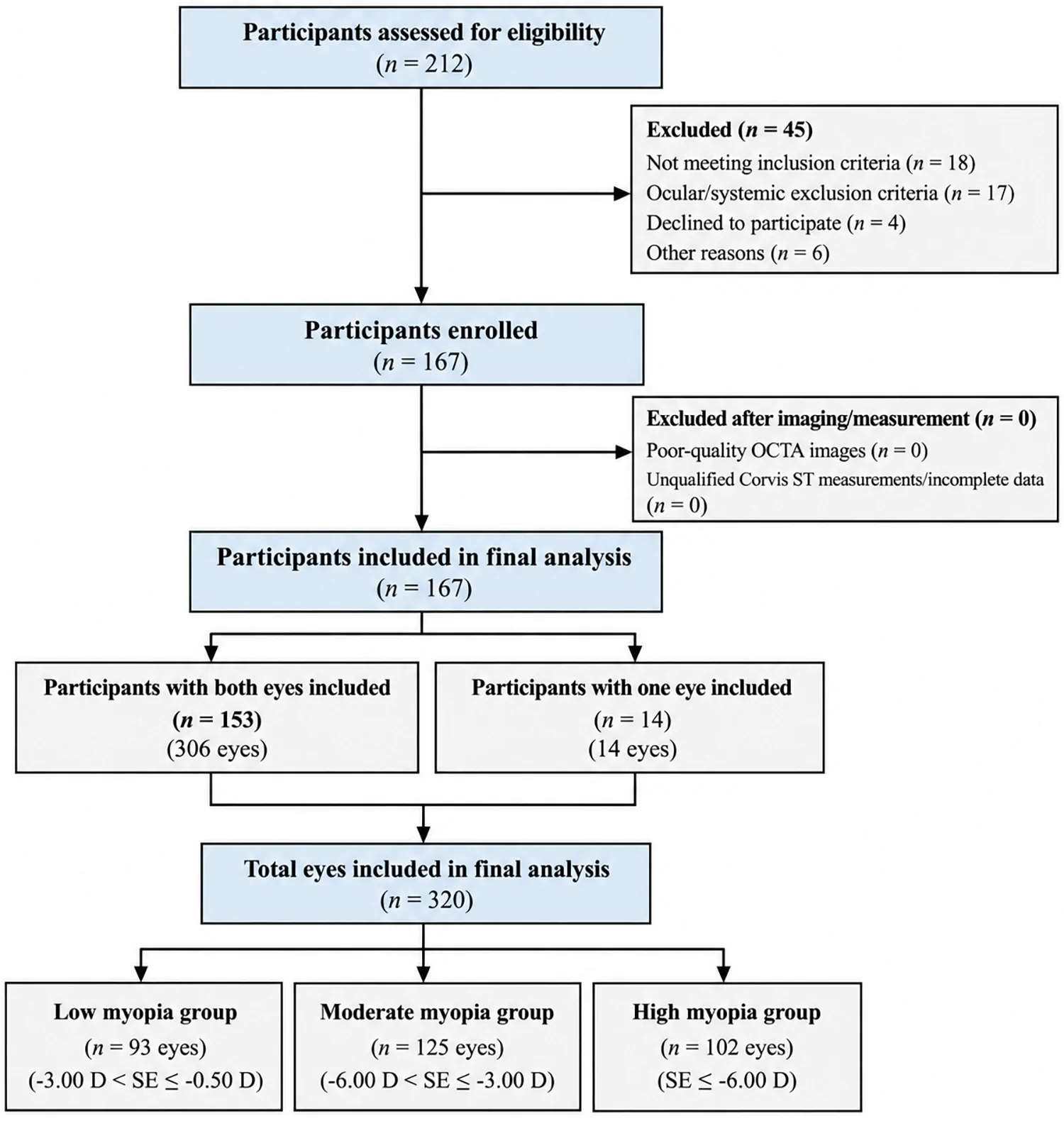
Flow diagram of participant recruitment, exclusions, and eyes included in the final analysis.

**Table 1:** Distribution characteristics of age, spherical equivalent (SE), axial length (AL), and intraocular pressure (IOP) among the three groups.

Group	Low myopia group	Moderate myopia group	High myopia group	*P*
Eyes, *n*	93	125	102	/
Gender (Male/Female), n	26/67	42/83	28/74	/
Age (years), mean ± SD	25.355 ± 3.185	26.616 ± 5.413	24.833 ± 4.088	/
SE (D), mean ± SD	−1.836 ± 0.696	−4.333 ± 0.851	−7.442 ± 1.331	<0.001^a^
Axial length (mm), mean ± SD	24.461 ± 0.686	25.381 ± 2.377	26.889 ± 0.946	<0.001^a^
IOP (mmHg), mean ± SD	15.289 ± 1.758	15.986 ± 2.234	16.330 ± 2.292	0.003^a^

The sample size (n) represents the number of eyes, as all variables in this table were summarized and analyzed at the eye level. SD, standard deviation.

a indicates a statistically significant difference between groups (*P* < 0.05).

### 3.2 Differences in corneal biomechanical properties among myopia groups

The statistical results and analyses of corneal biomechanical parameters among groups are detailed in Table 2. Significant intergroup differences were observed in PD, the SSI, SP-A1, and ARTh (all *P* < 0.05). Specifically, the low myopia group showed significantly lower PD (4.965 ± 0.524 mm vs. 5.162 ± 0.233 mm) and ARTh (568.28 ± 112.34 vs. 639.91 ± 220.28) values compared to the high myopia group (all *P* < 0.05). Both moderate and high myopia groups demonstrated higher SP-A1 values than the low myopia group (moderate vs. low: 104.538 ± 18.067 vs. 97.027 ± 15.540; high vs. low: 104.197 ± 16.247 vs. 97.027 ± 15.540; all *P* < 0.05). Additionally, the low and moderate myopia groups exhibited greater SSI values than the high myopia group (low vs. high 1.012 ± 0.168 vs. 0.886 ± 0.176, moderate vs. high 0.957 ± 0.180 vs. 0.886 ± 0.176; all *P* < 0.05). No statistically significant differences appeared in other corneal biomechanical parameters among groups (all *P* > 0.05).

**Table 2:** Statistical analysis of corneal biomechanical parameters among groups.

Parameter	Low myopia group	Moderate myopia group	High myopia group	P_ab_	P_ac_	P_bc_
DA（mm）	1.079 ± 0.090	1.086 ± 0.126	1.092 ± 0.999	0.956	0.795	0.966
AL1（mm）	2.299 ± 0.367	2.331 ± 0.380	2.310 ± 0.329	0.894	0.996	0.961
AV1（mm/s）	0.155 ± 0.226	0.153 ± 0.087	0.153 ± 0.057	0.992	0.929	0.984
AL2（mm）	1.917 ± 0.455	1.945 ± 0.391	1.850 ± 0.391	0.948	0.5	0.237
AV2（mm/s）	−0.279 ± 0.127	−0.265 ± 0.126	−0.281 ± 0.039	0.755	0.999	0.664
IRA（mm）	7.671 ± 1.210	7.567 ± 1.172	7.631 ± 0.909	0.868	0.992	0.963
PD（mm）	4.965 ± 0.524	5.063 ± 0.537	5.162 ± 0.233	0.330	0.010^a^	0.294
SSI	1.012 ± 0.168	0.957 ± 0.180	0.886 ± 0.176	0.068	<0.001^a^	0.008^a^
SP-A1	97.027 ± 15.540	104.538 ± 18.067	104.197 ± 16.247	0.004^a^	0.009^a^	0.998
ARTh	568.28 ± 112.34	583.62 ± 126.93	639.91 ± 220.28	0.862	0.006^a^	0.025^a^
DA Ratio	4.431 ± 0.867	4.289 ± 0.779	4.188 ± 0.597	0.432	0.075	0.680

DA, deformation amplitude; AL1, first applanation length; AV1, first applanation velocity; AL2, second applanation length; AV2, second applanation velocity; IRA, integrated radius; PD, peak distance; SSI, stress-strain index; SP-A1, stiffness parameter at first applanation; ARTh, ambrosio relational thickness-horizontal; DA ratio, deformation amplitude ratio; P_ab_, *P*-value for low vs. moderate myopia group comparison; P_ac_ *P*-value for low vs. high myopia group comparison; P_bc_ *P*-value for moderate vs. high myopia group comparison.

a indicates a statistically significant difference.

### 3.3 Differences in SVD of macular region among three myopia groups

Analysis of macular SVD across myopia groups is presented in Table 3. Linear mixed-effects model analyses showed significant intergroup differences in macular SVD in the IN and ON regions. Compared to the high myopia group, both low and moderate myopia groups showed significantly higher SVD values in the IN region (low vs. high: 15.812 ± 3.229 vs. 13.955 ± 3.971; moderate vs. high: 15.404 ± 3.170 vs. 13.955 ± 3.971; all *P* < 0.05). A similar trend was observed in the ON region (low vs. high: 18.986 ± 2.021 vs. 18.048 ± 2.341; moderate vs. high: 18.782 ± 2.011 vs. 18.048 ± 2.341; all *P* < 0.05). No statistically significant differences were found in other macular sectors (C, IS, II, IT, OS, OI, OT; all *P* > 0.05).

**Table 3:** Statistical analysis of macular SVD parameters by myopia severity.

Para-meter	Low myopia group	Moderate myopia group	High myopia group	P_ab_	P_ac_	P_bc_
C	5.493 ± 2.845	5.670 ± 2.999	5.611 ± 2.934	0.960	0.989	0.998
IS	15.320 ± 3.073	15.188 ± 3.020	14.612 ± 3.512	0.988	0.362	0.480
II	14.516 ± 3.998	14.482 ± 3.246	13.478 ± 3.673	1.000	0.115	0.095
IN	15.812 ± 3.229	15.404 ± 3.170	13.955 ± 3.971	0.775	0.001^a^	0.010^a^
IT	13.900 ± 3.517	13.819 ± 3.632	13.238 ± 4.079	0.999	0.554	0.592
OS	16.166 ± 2.642	16.224 ± 2.831	15.735 ± 2.653	0.998	0.622	0.462
OI	15.225 ± 2.820	15.199 ± 2.788	14.301 ± 3.078	1.000	0.078	0.060
ON	18.986 ± 2.021	18.782 ± 2.011	18.048 ± 2.341	0.872	0.012^a^	0.047^a^
OT	12.347 ± 3.796	12.519 ± 3.841	12.273 ± 3.800	0.983	0.999	0.949

C, central; IS, inner superior; II, inner inferior; IN, inner nasal; IT, inner temporal; OS, outer superior; OI, outer inferior; ON, outer nasal; OT, outer temporal; P_ab_, *P*-value for low vs. moderate myopia group comparison; P_ac_ *P*-value for low vs. high myopia group comparison; P_bc_ *P*-value for moderate vs. high myopia group comparison.

a indicates a statistically significant difference.

### 3.4 Differences in peripapillary SVD among three myopia groups

Peripapillary SVD across the three myopia groups is summarized in Table 4. Linear mixed-effects model analyses showed significant intergroup differences in the IN, IT, OS, OI, ON, and OT regions (all *P* < 0.05). In the IN and IT regions, SVD was significantly higher in both the low- and moderate-myopia groups than in the high-myopia group. For OS, OI, and ON SVD, significant differences were observed across all pairwise group comparisons, with lower values observed in groups with greater myopia severity. In the OT region, SVD was significantly lower in the high-myopia group than in both the low- and moderate-myopia groups. No statistically significant intergroup differences were observed in the IS or II regions.

**Table 4:** Statistical analysis of peripapillary SVD parameters by myopia severity.

Parameter	Low myopia group	Moderate myopia group	High myopia group	P_ab_	P_ac_	P_bc_
IS	19.030 ± 1.422	18.933 ± 1.860	18.402 ± 2.740	0.956	0.131	0.283
II	18.542 ± 2.291	18.770 ± 1.981	17.945 ± 3.205	0.945	0.328	0.111
IN	18.024 ± 2.337	17.861 ± 2.557	16.645 ± 3.790	0.969	0.011^a^	0.027^a^
IT	17.663 ± 2.973	17.238 ± 3.683	15.629 ± 4.890	0.975	0.010^a^	0.021^a^
OS	18.221 ± 1.885	16.749 ± 3.422	14.893 ± 4.237	0.003^a^	<0.001^a^	0.003^a^
OI	18.521 ± 1.738	16.657 ± 3.444	14.499 ± 4.856	0.008^a^	<0.001^a^	0.008^a^
ON	16.735 ± 2.362	14.465 ± 3.768	12.673 ± 4.699	<0.001^a^	<0.001^a^	0.002^a^
OT	15.883 ± 3.941	14.801 ± 5.074	13.052 ± 5.643	0.499	<0.001^a^	0.033^a^

P_ab_, *P*-value for low vs. moderate myopia group comparison; P_ac_ *P*-value for low vs. high myopia group comparison; P_bc_ *P*-value for moderate vs. high myopia group comparison.

a indicates a statistically significant difference.

### 3.5 Associations between corneal biomechanical parameters and retinal SVD

Associations between corneal biomechanical parameters and retinal SVD are presented in Table 5. After adjustment for AL, age, sex, and bIOP, the LMMs identified several significant associations. For macular SVD, AL2 was negatively associated with SVD in the IT (*β* = −1.359, 95% CI: −2.382 to −0.336; *P* = 0.009), OT (*β* = −1.090, 95% CI: −2.126 to −0.053; *P* = 0.039), and OS regions (*β* = −0.766, 95% CI: −1.505 to −0.027; *P* = 0.042). DA Ratio was positively associated with IT SVD (*β* = 0.685, 95% CI: 0.105 to 1.264; *P* = 0.021), whereas AV1 was negatively associated with IN SVD (*β* = −7.317, 95% CI: −14.304 to −0.330; *P* = 0.040). For peripapillary SVD, ARTh was negatively associated with SVD in the IN (*β* = −0.003, 95% CI: −0.006 to −0.0005; *P* = 0.023), OS (*β* = −0.005, 95% CI: −0.008 to −0.002; *P* = 0.003), OI (*β* = −0.004, 95% CI: −0.008 to −0.001; *P* = 0.015), ON (*β* = −0.004, 95% CI: −0.008 to −0.001; *P* = 0.016), and OT regions (*β* = −0.007, 95% CI: −0.011 to −0.002; *P* = 0.008). No other statistically significant associations were observed (all *P* > 0.05).

**Table 5:** Associations between corneal biomechanical parameters and retinal SVD based on LMMs.

Corneal biomechanical properties	Macular SVD	Peripapillary SVD	β	P	95%CI
AL2	IT	\	−1.358	0.009	−2.3815∼−0.3357
DA Ratio	IT	\	0.684	0.021	0.1053∼1.2644
AL2	OT	\	−1.089	0.039	−2.1264∼−0.0529
AV1	IN	\	−7.317	0.040	−14.3043∼−0.3303
AL2	OS	\	−0.766	0.042	−1.5050∼−0.0272
ARTh	\	IN	−0.003	0.023	−0.0062∼−0.0005
ARTh	\	OS	−0.005	0.003	−0.0083∼−0.0017
ARTh	\	OI	−0.004	0.015	−0.0078∼−0.0009
ARTh	\	ON	−0.004	0.016	−0.0082∼−0.0008
ARTh	\	OT	−0.006	0.008	−0.0113∼−0.0018

β represents the unstandardized regression coefficient derived from the linear mixed-effects model.

### 3.6 Associations between corneal biomechanical parameters and macular SVD according to myopia severity

To assess whether the associations between corneal biomechanical parameters and macular SVD differed according to myopia severity, interaction terms between each biomechanical parameter and myopia severity group were examined. Significant IRA × myopia severity interactions were observed for SVD in the central, II, IN, IT, and OT regions (all *P* for interaction < 0.05). A significant PD  ×  myopia severity interaction was also observed for central SVD (*P* for interaction = 0.023; Table 6).

**Table 6:** Interaction and stratified linear mixed-effects model analyses of the associations between corneal biomechanical parameters and macular SVD according to myopia severity.

Macular region	Parameter	Low myopia β (95% CI), P	Moderate myopia β (95% CI), P	High myopia β (95% CI), P	F	P for interaction
Center	IRA	0.357 (−0.150, 0.863), 0.165	−0.367 (−0.832, 0.097), 0.120	−0.466 (−1.160, 0.227), 0.185	3.885	0.022
Center	PD	0.065 (−1.202, 1.331), 0.920	0.541 (−0.523, 1.604), 0.316	−5.642 (−9.813, −1.472), 0.009	3.839	0.023
II	IRA	0.360 (−0.249, 0.970), 0.243	−0.483 (−0.991, 0.024), 0.062	−0.531 (−1.367, 0.305), 0.210	4.225	0.016
IN	IRA	0.532 (−0.002, 1.065), 0.051	−0.420 (−0.911, 0.070), 0.092	−0.020 (−0.943, 0.903), 0.966	3.892	0.021
IT	IRA	0.400 (−0.228, 1.028), 0.208	−0.610 (−1.167, −0.054), 0.032	−0.828 (−1.777, 0.121), 0.086	4.430	0.013
OT	IRA	0.307 (−0.376, 0.990), 0.374	−0.556 (−1.138, 0.027), 0.061	−1.151 (−2.028, −0.274), 0.011	4.416	0.013

β represents the unstandardized regression coefficient estimated from the linear mixed-effects model. F represents the F statistic from the Type III test of fixed effects for the biomechanical parameter × myopia severity group interaction in the full-sample model; P for interaction represents the corresponding *P* value.

Stratified analyses further characterized these interaction patterns. No significant associations were observed in the low-myopia group. In the moderate-myopia group, IRA was negatively associated with IT SVD (*β* = −0.610, *P* = 0.032), whereas in the high-myopia group, IRA was negatively associated with OT SVD (*β* = −1.151, *P* = 0.011) and PD was negatively associated with central SVD (*β* = −5.642, *P* = 0.009). Additional subgroup-specific associations were observed for AL2 and SSI; however, their interaction terms were not statistically significant, indicating no evidence that these associations differed across myopia severity groups.

### 3.7 Associations between corneal biomechanical parameters and peripapillary SVD according to myopia severity

For peripapillary SVD, interaction analysis identified a significant interaction between IRA and myopia severity in the OS region (F = 4.242, *P* for interaction = 0.015, Table 7), suggesting that the association between IRA and OS SVD differed across myopia severity groups. However, stratified analyses showed that the corresponding within-group associations did not reach statistical significance in any of the three groups.

**Table 7:** Interaction and stratified analyses of the associations between corneal biomechanical parameters and peripapillary SVD according to myopia severity.

Macular region	Parameter	Low myopia β (95% CI), P	Moderate myopia β (95% CI), P	High myopia β (95% CI), P	F	P for interaction
OS	IRA	0.011 (−0.323, 0.344), 0.950	0.426 (−0.088, 0.939), 0.103	−0.450 (−1.568, 0.669), 0.426	4.242	0.015
ON	AV1	30.707 (2.100, 59.315), 0.036	−1.399 (−8.996, 6.198), 0.715	−22.189 (−101.037, 56.659), 0.577	0.376	0.687
II	SSI	1.344 (−1.617, 4.305), 0.369	−2.621 (−4.755, −0.487), 0.017	1.150 (−2.307, 4.606), 0.501	2.715	0.068
OS	ARTh	0.0024 (−0.0013, 0.0062), 0.201	−0.0084 (−0.0140, −0.0029), 0.003	−0.0036 (−0.0097, 0.0026), 0.249	2.426	0.090
OT	ARTh	0.0022 (−0.0060, 0.0104), 0.589	−0.0091 (−0.0176, −0.0006), 0.036	−0.0069 (−0.0154, 0.0015), 0.107	2.035	0.133
OT	AL2	−0.588 (−2.456, 1.280), 0.533	−0.629 (−3.037, 1.780), 0.604	−3.335 (−6.490, −0.180), 0.039	0.327	0.721

β represents the unstandardized regression coefficient estimated from the linear mixed-effects model. F represents the F statistic from the Type III test of fixed effects for the biomechanical parameter × myopia severity group interaction in the full-sample model; P for interaction represents the corresponding *P* value.

Stratified analyses additionally identified several subgroup-specific associations. In the low-myopia group, AV1 was positively associated with ON SVD (*β* = 30.707, 95% CI: 2.100 to 59.315; *P* = 0.036). In the moderate-myopia group, SSI was negatively associated with II SVD (*β* = −2.621, 95% CI: −4.755 to −0.487; *P* = 0.017), while ARTh was negatively associated with OS SVD (*β* = −0.0085, 95% CI: −0.0140 to −0.0029; *P* = 0.003) and OT SVD (*β* = −0.0091, 95% CI: −0.0176 to −0.0006; *P* = 0.036). In the high-myopia group, AL2 was negatively associated with OT SVD (*β* = −3.335, 95% CI: −6.490 to −0.180; *P* = 0.039). However, the corresponding interaction terms for these subgroup-specific associations were not statistically significant, providing no evidence that these associations differed significantly across myopia severity groups.

## 4 Discussion

In this cross-sectional study, we evaluated differences in corneal biomechanical parameters and retinal SVD among eyes with different myopia severity and examined their adjusted associations. After accounting for inter-eye correlation, significant intergroup differences were observed in PD, SSI, SP-A1, and ARTh, although these parameters did not change in a uniform direction. Regional differences in macular and peripapillary SVD were also identified. After adjustment for AL, age, sex, and bIOP, selected Corvis ST parameters remained associated with regional SVD. Formal interaction analyses further indicated that some associations involving IRA and PD differed according to myopia severity. These results suggest a potential relationship between ocular dynamic response characteristics and retinal microvascular measurements, but they do not establish causality or longitudinal changes during myopia progression.

It is important to recognize that Corvis ST-derived parameters reflect different aspects of the dynamic ocular response to an air-puff stimulus rather than interchangeable direct measures of intrinsic corneal stiffness. In the present study, SP-A1 was higher in the moderate- and high-myopia groups, whereas SSI was lower in the high-myopia group. This apparently discordant pattern should not be interpreted simply as greater or lower corneal stiffness. SP-A1 reflects the corneal response at first applanation and may be influenced by IOP and ocular geometry (14). whereas SSI is an algorithm-derived index intended to characterize corneal material stress-strain behavior. Therefore, the opposite changes in SP-A1 and SSI may reflect their sensitivity to different components of ocular biomechanical behavior rather than contradictory changes in a single biomechanical property. Similarly, PD and ARTh characterize different aspects of the dynamic deformation response and corneal thickness profile. Accordingly, the observed findings are more appropriately interpreted as parameter-specific differences in Corvis ST-derived dynamic ocular response characteristics (25).

Regional differences in SVD were observed in both the macular and peripapillary regions. Macular SVD differed among the myopia groups in the IN and ON regions, whereas peripapillary SVD differed in the IN, IT, OS, OI, ON, and OT regions. In most of these regions, lower SVD was observed in eyes with greater myopia severity. Wang et al.. reported significantly decreased macular SVD in high myopia, with clear associations with refractive and ocular factors (26). Similarly, Yang et al.. demonstrated differences in macular and peripapillary SVD associated with axial elongation in young Chinese adults (27), and other studies have also reported associations between AL and regional retinal vascular measurements in myopic eyes (28). Nevertheless, OCTA-derived SVD is a quantitative imaging parameter and does not by itself demonstrate vascular degeneration, impaired perfusion, or histopathological damage. Accordingly, the present findings should be described as regional differences or reductions in SVD rather than retinal microvascular degeneration. In addition, if ocular magnification correction was not applied, AL-related differences in image scaling may have influenced the quantitative SVD measurements, particularly in highly myopic eyes.

After adjustment for AL, age, sex, and bIOP, several associations between Corvis ST parameters and regional SVD remained statistically significant. For macular SVD, AL2 was negatively associated with SVD in the IT, OT, and OS regions, DA Ratio was positively associated with IT SVD, and AV1 was negatively associated with IN SVD. For peripapillary SVD, ARTh was negatively associated with SVD in the IN, OS, OI, ON, and OT regions. Previous studies have suggested that anterior ocular biomechanical characteristics may be related to posterior segment vascular measurements, although the available evidence remains limited and has largely been obtained from specific populations, such as children or patients with glaucoma and myopia (22, 23). The present study extends these observations to young adults with different degrees of myopia. However, because AL2, AV1, DA Ratio, and ARTh describe different features of the corneal dynamic response or thickness profile, these associations should not be interpreted as evidence of a single relationship between “corneal stiffness” and retinal SVD.

Formal interaction analyses provided evidence that selected associations differed according to myopia severity. Significant IRA × myopia severity interactions were identified for SVD in the central, II, IN, IT, and OT macular regions, and a significant PD × myopia severity interaction was found for central SVD. Stratified analyses showed no significant associations in the low-myopia group. IRA was negatively associated with IT SVD in the moderate-myopia group and with OT SVD in the high-myopia group, whereas PD was negatively associated with central SVD in the high-myopia group. These findings indicate statistical heterogeneity in selected associations across myopia groups. However, because the study was cross-sectional and the severity groups consisted of different participants, these results cannot establish temporal changes or distinct pathophysiological stages during myopia progression.

For peripapillary SVD, a significant IRA×myopia severity interaction was observed in the OS region, although the corresponding within-group associations did not reach statistical significance in any of the three groups. This pattern may occur because the interaction test evaluates differences between group-specific slopes rather than the significance of each individual slope. Therefore, the result suggests possible between-group heterogeneity but does not establish a statistically significant or clinically meaningful association within any particular myopia group. Additional associations were identified in the stratified analyses for AV1, SSI, ARTh, and AL2; however, their corresponding interaction terms were not significant. A significant association in one subgroup and a nonsignificant association in another does not necessarily indicate that the associations differ between groups. These subgroup-specific findings should therefore be interpreted as exploratory rather than as evidence of severity-dependent relationships.

The mechanisms underlying the observed relationships remain uncertain. One possible explanation is that corneal dynamic response parameters and retinal SVD are both influenced by changes in ocular geometry and the corneoscleral envelope. Axial elongation may be accompanied by scleral and retinal tissue remodeling and redistribution of mechanical stress, potentially affecting both anterior ocular deformation and posterior segment vascular measurements (27, 28). Previous studies have also identified genetic and molecular pathways potentially involved in myopia-related ocular remodeling and vascular regulation (29–36). However, genetic variants, extracellular-matrix composition, cytokines, and growth factors were not measured in the present study. These pathways therefore represent possible hypotheses rather than mechanisms demonstrated by our findings. Similarly, although SP-A1 may be influenced by IOP-related loading (14), systemic blood pressure and ocular perfusion pressure were not measured. Therefore, the present study cannot determine whether IOP-related hemodynamic changes contributed to the observed SVD patterns. Notably, after adjustment for AL, age, sex, and bIOP, SP-A1 was not significantly associated with macular or peripapillary SVD in any region. Accordingly, the present findings do not support an independent association between SP-A1 and regional SVD.

This study has several strengths. Corneal biomechanical and retinal microvascular measurements were obtained in the same cohort, allowing their associations to be evaluated at the eye level. The use of LMMs accounted for the correlation between fellow eyes while retaining all eligible observations. Adjustment for AL, age, sex, and bIOP reduced the influence of several important potential confounders. Moreover, formal interaction testing provided a more appropriate assessment of whether the associations differed according to myopia severity than comparisons based solely on subgroup-specific statistical significance.

Several limitations should be acknowledged. First, the cross-sectional design precludes assessment of temporal changes during myopia progression and does not permit causal inference. The present study therefore cannot determine whether differences in corneal dynamic response precede, accompany, or follow alterations in retinal SVD. Second, restricted by software analytical capabilities, we employed the ETDRS method for peripapillary analysis rather than the more specialized GH partitioning approach. Third, residual confounding remains possible because CCT, systemic blood pressure, ocular perfusion pressure, OCTA signal strength, and other ocular or systemic factors were not included in the models. Fourth, if AL-based ocular magnification correction was not applied, differences in image scaling may have affected the OCTA measurements. Fifth, automated image processing may not eliminate all motion artifacts or segmentation errors. Sixth, the sample size within each myopia group may have limited the precision of the stratified estimates and interaction analyses. In addition, no additional AL-based ocular magnification correction was applied during OCTA post-processing. Because axial elongation may affect the actual retinal area represented by a nominal scan size, particularly in highly myopic eyes, some magnification-related bias in regional SVD measurements cannot be excluded. Although AL was included as a covariate in the multivariable association models, this statistical adjustment does not fully substitute for image-level magnification correction. Finally, numerous associations were examined across multiple parameters and retinal regions. Particularly in the absence of comprehensive multiple-testing correction, these findings should be considered exploratory and require independent validation.

In summary, corneal dynamic response parameters and retinal SVD showed regional and parameter-specific differences across myopia severity groups. After accounting for inter-eye correlation and adjusting for AL, age, sex, and bIOP, selected Corvis ST parameters remained associated with regional macular or peripapillary SVD. Formal interaction analyses further suggested that some associations involving IRA and PD differed according to myopia severity. These findings support a potential relationship between anterior ocular biomechanical characteristics and retinal microvascular measurements, but do not establish causal mechanisms, longitudinal progression, diagnostic or prognostic performance, or clinical biomarker utility. No predictive model, clinically applicable threshold, or incremental predictive value beyond established factors such as AL was evaluated in the present study. Longitudinal studies incorporating standardized OCTA quality control, ocular magnification correction, appropriate control of multiple testing, and independent validation are required to determine the reproducibility and potential clinical relevance of these associations.

## 5 Conclusions

In young adults with myopia, corneal biomechanical parameters and retinal SVD showed significant regional differences across myopia severity groups. After accounting for inter-eye correlation and adjusting for AL, age, sex, and bIOP, several corneal biomechanical parameters were associated with regional macular and peripapillary SVD, with some associations differing according to myopia severity. These findings suggest a potential relationship between corneal biomechanical characteristics and retinal microvascular alterations in myopic eyes. Further longitudinal studies are needed to clarify the temporal and potential mechanistic relationships underlying these associations.

## Data Availability

The raw data supporting the conclusions of this article will be made available by the authors, without undue reservation.

## References

[1] Sankaridurg P Tahhan N Kandel H Naduvilath T Zou H Frick KD, et al.. IMI impact of myopia. Invest Ophthalmol Vis Sci. (2021) 62(5):2. 10.1167/iovs.62.5.2 33909036 PMC8083082

[2] Wolffsohn JS Calossi A Cho P Gifford K Jones L Jones D, et al.. Global trends in myopia management attitudes and strategies in clinical practice— 2019 update. Cont Lens Anterior Eye. (2020) 43(1):9–17. 10.1016/j.clae.2019.11.002 31761738

[3] Holden BA Fricke TR Wilson DA Jong M Naidoo KS Sankaridurg P, et al.. Global prevalence of myopia and high myopia and temporal trends from 2000 through 2050. Ophthalmology. (2016) 123(5):1036–42. 10.1016/j.ophtha.2016.01.006 26875007

[4] Liang J Pu Y Chen J Liu M Ouyang B Jin Z, et al.. Global prevalence, trend and projection of myopia in children and adolescents from 1990 to 2050: a comprehensive systematic review and meta-analysis. Br J Ophthalmol. (2025) 109(3):362–71. 10.1136/bjo-2024-325427 39317432

[5] Shah R Vlasak N Evans BJW. High myopia: reviews of myopia control strategies and myopia complications. Ophthalmic Physiol Opt. (2024) 44(6):1248–60. 10.1111/opo.13366 39082137

[6] Bullimore MA Brennan NA. Myopia control: why each diopter matters. Optom Vis Sci. (2019) 96(6):463–5. 10.1097/OPX.0000000000001367 31116165

[7] Ohno-Matsui K Kawasaki R Jonas JB Cheung CMG Saw S-M Verhoeven VJM, et al.. International photographic classification and grading system for myopic maculopathy. Am J Ophthalmol. (2015) 159(5):877–83.e7. 10.1016/j.ajo.2015.01.022 25634530

[8] Xin Y Kang BS Zheng YP Shan SW Kee CS Tan Y. Biophysical properties of corneal cells reflect high myopia progression. Biophys J. (2021) 120(16):3498–507. 10.1016/j.bpj.2021.05.010 34022236 PMC8391025

[9] Chen Q Song K Cun Q Yang W Tao Y Zhong H, et al.. Differences in macular structure and microvasculature between high myopia and non-high myopia. Front Med (Lausanne). (2025) 12:1645015. 10.3389/fmed.2025.1645015 40933577 PMC12417401

[10] Czepita D Mojsa A Ustianowska M Czepita M Lachowicz E. Reading, writing, working on a computer or watching television, and myopia. Klin Oczna. (2010) 112(10-12):293–5. 21469524

[11] Tedja MS Haarman AEG Meester-Smoor MA Kaprio J Mackey DA Guggenheim JA, et al.. IMI— myopia genetics report. Invest Ophthalmol Vis Sci. (2019) 60(3):M89–m105. 10.1167/iovs.18-25965 30817828 PMC6892384

[12] Li DL Lanca C Zhang XJ Grzybowski A He XG Pan CW. Spatial frequency of environments and myopia: a systematic review on associated evidence and underlying mechanisms. Acta Ophthalmol. (2025) 103(8):909–18. 10.1111/aos.17437 39754355

[13] Monleón CH-B Navarro DG Plata CS Rufas IM Pérez EF Merino JY Digital devices and the myopia epidemic in children: short- and long-term effects of use and strategies to manage ocular symptoms. Acta Ophthalmol (Copenh). (2025):103. 10.1111/aos.17164

[14] Chen H Wu S Tian L Li Y Hong J Wang Y, et al.. Intraocular pressure measurement and association with corneal biomechanics in patients underwent Descemet’s stripping with endothelial keratoplasty: a comparative study. Front Med (Lausanne). (2024) 11:1384694. 10.3389/fmed.2024.1384694 39071083 PMC11272548

[15] Wu Y Wang Y. Detailed distribution of corneal epithelial thickness and correlated characteristics measured with SD-OCT in myopic eyes. J Ophthalmol. (2017) 2017:1018321. 10.1155/2017/1018321 28607770 PMC5457757

[16] Kim BJ Ryu IH Lee JH Kim SW. Correlation of sex and myopia with corneal epithelial and stromal thicknesses. Cornea. (2016) 35(8):1078–83. 10.1097/ICO.0000000000000850 27227393

[17] Lin CR Toychiev A Ablordeppey RK Srinivas M Benavente-Perez A. Sustained retinal defocus increases the effect of induced myopia on the retinal astrocyte template. Cells. (2024) 13(7):595. 10.3390/cells13070595 38607034 PMC11011523

[18] Chen C-Y Huang EJ-C Kuo C-N Wu P-L Chen C-L Wu P-C, et al.. The relationship between age, axial length and retinal nerve fiber layer thickness in the normal elderly population in Taiwan: the Chiayi eye study in Taiwan. PLoS One. (2018) 13(3):e0194116. 10.1371/journal.pone.0194116 29522558 PMC5844564

[19] Swiatczak B Feldkaemper M Schaeffel F. Changes in fundus reflectivity during myopia development in chickens. Biomed Opt Express. (2019) 10(4):1822–40. 10.1364/BOE.10.001822 31086706 PMC6485001

[20] Ablordeppey RK Nieu R Lin CR Benavente-Perez A. Early alterations in inner-retina neural and glial saturated responses in lens-induced myopia. Transl Vis Sci Technol. (2024) 13(4):16. 10.1167/tvst.13.4.16 38591944 PMC11008749

[21] Wong CW Teo YCK Tsai STA Ting SWD Yeo YSI Wong WKD, et al.. Characterization of the choroidal vasculature in myopic maculopathy with optical coherence tomographic angiography. Retina. (2019) 39(9):1742–50. 10.1097/IAE.0000000000002233 29952919

[22] Ohn K Jung Y Park HL. Differential effects of corneal biomechanics on superficial and deep vessel density and their association with central visual function in glaucoma patients with myopia. J Clin Med. (2025) 14(18):6515. 10.3390/jcm14186515 41010718 PMC12470511

[23] Lim L Cheung N Gazzard G Chan YH Wong TY Saw SM. Corneal biomechanical properties and retinal vascular caliber in children. Invest Ophthalmol Vis Sci. (2009) 50(1):121–5. 10.1167/iovs.08-2352 18775868

[24] Rosenfeld PJ Durbin MK Roisman L Zheng F Miller A Robbins G, et al.. ZEISS angioplex™ spectral domain optical coherence tomography angiography: technical aspects. Dev Ophthalmol. (2016) 56:18–29. 10.1159/000442773 27023249

[25] Miki A Maeda N Asai T Ikuno Y Nishida K. Measurement repeatability of the dynamic scheimpflug analyzer. Jpn J Ophthalmol. (2017) 61(6):433–40. 10.1007/s10384-017-0534-9 28983780

[26] Wang T Li H Zhang R Yu Y Xiao X Wu C. Evaluation of retinal vascular density and related factors in youth myopia without maculopathy using OCTA. Sci Rep. (2021) 11(1):15361. 10.1038/s41598-021-94909-8 34321564 PMC8319333

[27] Yang D Cao D Zhang L Yang C Lan J Zhang Y, et al.. Macular and peripapillary vessel density in myopic eyes of young Chinese adults. Clin Exp Optom. (2020) 103(6):830–7. 10.1111/cxo.13047 32052475

[28] Guo Y Pang Y Kang YJ Zhang X Zhang H Zhang G, et al.. Correlations among peripapillary vasculature, macular superficial capillaries, and eye structure in healthy and myopic eyes of Chinese young adults (STROBE). Medicine (Baltimore). (2020) 99(37):e22171. 10.1097/MD.0000000000022171 32925785 PMC7489708

[29] Wang YY Chen LJ Tham CC Yam JC Pang CP. Genes for childhood myopia. Asia Pac J Ophthalmol (Phila). (2025) 14(1):100139. 10.1016/j.apjo.2025.100139 39814143

[30] Gong Q Janowski M Xie M Yang G Liu L. Rasgrf1 mRNA expression in myopic eyes of Guinea pigs. Clin Exp Optom. (2017) 100(2):174–8. 10.1111/cxo.12476 27723119

[31] Fan Q Barathi VA Cheng C-Y Zhou X Meguro A Nakata I, et al.. Genetic variants on chromosome 1q41 influence ocular axial length and high myopia. PLoS Genet. (2012) 8(6):e1002753. 10.1371/journal.pgen.1002753 22685421 PMC3369958

[32] Li FF Lu SY Tang SM Kam KW Pancy O S T Yip WWK, et al.. Genetic associations of myopia severities and endophenotypes in children. Br J Ophthalmol. (2021) 105(8):1178–83. 10.1136/bjophthalmol-2020-316728 32816751

[33] Drzyzga Ł Śpiewak D Dorecka M Wyględowska-Promieńska D. Available therapeutic options for corneal neovascularization: a review. Int J Mol Sci. (2024) 25(10):5479. 10.3390/ijms25105479 38791518 PMC11121997

[34] Srejovic JV Muric MD Jakovljevic VL Srejovic IM Sreckovic SB Petrovic NT, et al.. Molecular and cellular mechanisms involved in the pathophysiology of retinal vascular disease-interplay between inflammation and oxidative stress. Int J Mol Sci. (2024) 25(21):11850. 10.3390/ijms252111850 39519401 PMC11546760

[35] Chikama T Liu C-Y Meij JTA Hayashi Y Wang I-J Yang L, et al.. Excess FGF-7 in corneal epithelium causes corneal intraepithelial neoplasia in young mice and epithelium hyperplasia in adult mice. Am J Pathol. (2008) 172(3):638–49. 10.2353/ajpath.2008.070897 18276784 PMC2258268

[36] McAvoy JW Chamberlain CG. Growth factors in the eye. Prog Growth Factor Res. (1990) 2(1):29–43. 10.1016/0955-2235(90)90008-8 2104273

